# Genetic polymorphisms in *PTPN22*, *PADI-4*, and *CTLA-4 *and risk for rheumatoid arthritis in two longitudinal cohort studies: evidence of gene-environment interactions with heavy cigarette smoking

**DOI:** 10.1186/ar2421

**Published:** 2008-05-07

**Authors:** Karen H Costenbader, Shun-Chiao Chang, Immaculata De Vivo, Robert Plenge, Elizabeth W Karlson

**Affiliations:** 1 Division of Rheumatology, Immunology, and Allergy, Department of Medicine, Brigham and Women's Hospital, Harvard Medical School, Boston, Massachusetts 02115, USA; 2 Channing Laboratory, Department of Medicine, Brigham and Women's Hospital, Harvard Medical School, 75 Francis Street Boston, Massachusetts 02115, USA; 3 Department of Epidemiology, Harvard School of Public Health, 677 Huntington Avenue, Boston, Massachusetts 02115, USA

## Abstract

**Introduction:**

*PTPN22*, *PADI-4*, and *CTLA-4 *have been associated with risk for rheumatoid arthritis (RA). We investigated whether polymorphisms in these genes were associated with RA in Caucasian women included in two large prospective cohorts, adjusting for confounding factors and testing for interactions with smoking.

**Methods:**

We studied RA risk associated with *PTPN22 *(rs2476601), *PADI-4 *(rs2240340), and *CTLA-4 *(rs3087243) in the Nurses' Health Study (NHS) and NHSII. Participants in NHS were aged 30 to 55 years at entry in 1976; those in NHSII were aged 25 to 42 years at entry in 1989. We confirmed incident RA cases through to 2002 in NHS and to 2003 in NHSII by questionnaire and medical record review. We excluded reports not confirmed as RA. In a nested case-control design involving participants for whom there were samples for genetic analyses (45% of NHS and 25% of NHSII), each incident RA case was matched to a participant without RA by year of birth, menopausal status, and postmenopausal hormone use. Genotyping was performed using Taqman single nucleotide polymorphism allelic discrimination on the ABI 7900 HT (Applied Biosystems, 850 Lincoln Centre Drive, Foster City, CA 94404 USA) with published primers. Human leukocyte antigen shared epitope (*HLA-SE*) genotyping was performed at high resolution. We employed conditional logistic regression analyses, adjusting for smoking and reproductive factors. We tested for additive and multiplicative interactions between each genotype and smoking.

**Results:**

A total of 437 incident RA cases were matched to healthy female control individuals. Mean (± standard deviation) age at RA diagnosis was 55 (± 10), 57% of RA cases were rheumatoid factor (RF) positive, and 31% had radiographic erosions at diagnosis. *PTPN22 *was associated with increased RA risk (pooled odds ratio in multivariable dominant model = 1.46, 95% confidence interval [CI] = 1.02 to 2.08). The risk was stronger for RF-positive than for RF-negative RA. A significant multiplicative interaction between *PTPN22 *and smoking for more than 10 pack-years was observed (*P *= 0.04). *CTLA-4 *and *PADI-4 *genotypes were not associated with RA risk in the pooled results (pooled odds ratios in multivariable dominant models: 1.27 [95% CI = 0.88 to 1.84] for *CTLA-4 *and 1.04 [95% CI = 0.77 to 1.40] for *PADI-4*). No gene-gene interaction was observed between *PTPN22 *and *HLA-SE*.

**Conclusion:**

After adjusting for smoking and reproductive factors, *PTPN22 *was associated with RA risk among Caucasian women in these cohorts. We found both additive and multiplicative interactions between *PTPN22 *and heavy cigarette smoking.

## Introduction

Rheumatoid arthritis (RA) is a systemic autoimmune disease that is characterized by chronic, destructive, and debilitating arthritis. The most common inflammatory arthritis, it affects approximately 1% of the population [1]. The etiology of RA is unknown, but it is presumed that environmental factors trigger its development in the genetically predisposed. The strongest genetic risk factor, which is responsible for approximately 30% of the genetic contribution to the development of RA, is human leukocyte antigen (HLA) type [2,3]. The HLA-shared epitope (SE) is the strongest known genetic risk factor for RA, with two copies associated with a relative risk of 5 to 6 in Caucasians [4-6]. However, RA, like many complex human diseases, is polygenic in origin. It is likely that many other genes, both inside and outside the HLA region, contribute to disease predisposition [5,7-10]. RA genetic studies have reported polymorphisms *PTPN22 *(R620W, rs2476601) [9,11-16], *CTLA-4 *(CT60, rs3087243) [17,18], and *PADI-4 *(PADI4_94, rs2240340) [8] to be associated with increased risk for RA. More recently, polymorphisms in or near the genes encoding *STAT4 *[19], *TRAF1-C5 *[20], *TNF-AIP2 *[16], and *IL2RA *[16] have been reported in whole-genome scans. The non-HLA genetic polymorphism that has been most strongly replicated across multiple independent studies is *PTPN22 *[9,11-14,16,21-24]. This missense allele (C→T) has been associated in past studies with an odds ratio (OR) for RA of approximately 1.8 [9], and it appears to carry greater risk for autoantibody-positive RA [12,24,25]. Homozygotes for the variant T allele are at greatest risk for RA (OR = 4.6) [12]. This polymorphism has also been associated with increased risk for type I diabetes [26,27] and systemic lupus erythematosus [11,13]. The associations of *CTLA-4 *and *PADI-4 *polymorphisms with RA risk have been less well replicated [17,18,28]. In a large pooled replication study, *CTLA4 *CT60 polymorphism was associated with anti-cyclic citrullinated peptide antibody (anti-CCP)-positive RA [25].

Twin studies conducted in the UK and Finland have estimated that 50% to 60% of the variation in RA susceptibility is accounted for by genetic factors [29], leaving 40% to 50% probably due to environmental exposures. Cigarette smoking is the best established environmental risk factor for RA, with risk increasing in proportion to duration and intensity of exposure [30-35]. Case-control studies conducted in Sweden, Holland, and North America have identified an interaction between presence of the *HLA*-*SE *alleles and cigarette smoking in determining RA risk, in particular that of anti-CCP-positive RA [36-38]. Female reproductive factors such as early age at menarche, irregular menses, and use of postmenopausal hormones have also been related to increased RA risk, and prolonged duration of breast-feeding was found to be protective against development of RA in the Nurses' Health Study (NHS) [39-41].

We aimed to validate previous findings of increased risk for RA associated with polymorphisms in the *PTPN22*, *PADI-4 *and *CTLA-4 *genes, and to assess whether of behavioral and reproductive factors that are known to be associated with RA risk influence these findings. We also investigated potential additive and multiplicative interactions between each of these polymorphisms and the presence of the *HLA-SE*. To do this, we conducted a case-control study nested within the NHS and NHSII; those studies include two large cohorts of women, who were followed closely over many years for behavioral and reproductive factors before the onset of disease.

## Materials and methods

### Study population

The NHS includes a prospective cohort of 121,700 female nurses, aged 30 to 55 years in 1976 when the study began. The NHSII was established in 1989, when 116,608 female nurses aged 25 to 42 years completed a baseline questionnaire about their medical histories and lifestyles. Ninety-four per cent of the NHS participants from 1976 to 2002, and 95% of NHSII participants from 1989 to 2003 have remained in active follow up (5% to 6% no longer respond to questionnaires and have not been confirmed as dead). All aspects of this study were approved by the Partners' HealthCare Institutional Review Board.

### Identification of rheumatoid arthritis

As previously described [35], we employed a two-stage procedure in which all nurses who self-reported any connective tissue disease received a screening questionnaire for connective tissue disease symptoms [42], and – if positive – a detailed medical record review for American College of Rheumatology (ACR) classification criteria for RA [43], in order to identify and validate incident cases of RA. The presence or absence of rheumatoid factor (RF) and other features of RA was based on medical record review. Those in whom four of the seven ACR criteria were documented in the medical record were considered to have definite RA. For this nested case-control study, we also included a small number of women (*n* = 14) with three documented ACR criteria for RA, a diagnosis of RA by their physician, and agreement by two rheumatologists on the diagnosis of RA.

### Population for analysis

We excluded prevalent RA cases diagnosed before the cohort was assembled, nonresponders, and women who reported any connective tissue disease that was not subsequently confirmed to be RA by medical record review. Women were censored when they failed to respond to any subsequent biennial questionnaires. Among the women in each cohort who had provided a sample for genetic analyses, each participant with confirmed incident RA was matched by year of birth, menopausal status, and postmenopausal hormone use to a healthy woman in the same cohort without RA. To minimize population stratification, and given that most cohort participants are Caucasian, we limited the analyses to Caucasian matched pairs of women. In 1992 (NHS) and 1989 (NHSII), all participants were asked to provide data concerning their own racial backgrounds in more detailed categories. Of the Caucasian women in NHS and NHSII included in this analysis, 2% reported pure Scandinavian heritage, 15% reported pure Southern European, and 83% reported other or mixed Caucasian backgrounds. There were no significant differences in the distributions of these ethnicities between cases and controls (χ^2 ^with two degrees of freedom, *P *= 0.30).

### Blood sampling

From 1989 to 1990, 32,826 (27%) NHS participants aged 43 to 70 years agreed to provide blood samples for future NHS studies. Between 1996 and 1999, 29,613 (25%) of the women included in the NHSII cohort (aged 32 to 52 years at that time) also agreed to have their blood drawn for future investigations. All samples were collected in heparinized tubes and sent to us by overnight courier in chilled containers. On receipt, the blood samples were centrifuged, aliquoted, and stored in liquid nitrogen freezers at -70°F (-57°C). The demographic and exposure characteristics of the NHS and NHSII participants who provided blood samples were found to be very similar to those of the overall cohorts [44,45].

### DNA extraction from blood

DNA was extracted from buffy coats from 96 samples in 3 to 4 hours. A volume of 50 μl of buffy coat was diluted with 150 μl phosphate-buffered saline and processed using the QIAmp™ (QIAGEN Inc., Chatsworth, CA, USA) 96-spin blood kit protocol. The protocol entails adding protease, the sample, and lysis buffer to 96-well plates. The plates are then mixed and incubated at 158°F (70°C), before adding ethanol and transferring the samples to columned plates. The columned plates are then centrifuged and washed with buffer. Adding elution buffer and centrifuging elutes the DNA. The average yield from 50 μl of buffy coat (based on 1,000 samples) is 5.5 μg with a standard deviation of 2.2 (range 2.0 to 16.4). These methods are semiautomated using a Qiagen 8000 robot to increase throughput and decrease manual pipetting errors.

### Buccal cell collection method and DNA extraction in NHS

Forty thousand women in NHS who did not give blood in 1989 to 1990 were asked to give a buccal cell sample in 2002. To date we have collected an additional 21,733 buccal cell samples (18% of the NHS cohort). A collection kit was sent to participants, consisting of instructions for the buccal cell collection and the necessary supplies (a small bottle of mouthwash, a plastic cup with a screwtop cap, a ziplock plastic bag and absorbent sheet, and a stamped, self-addressed bubble envelope), as well as an informed consent form. Participants were instructed to fill the cup with mouthwash, swish the mouthwash in their mouth vigorously, and then spit back into the cup. Returned samples were processed using ReturPureGene DNA Isolation Kit (Gentra Systems, Minneapolis, MN, USA) to extract genomic DNA from human cheek cells. The extracted DNA was archived in liquid nitrogen freezers using specific tracking software. The average DNA recovery from these specimens measured using PicoGreen was 59 ng/μl.

### Whole-genome amplification

For all genomic DNA samples, an aliquot was put through a whole-genome amplification protocol using the GenomPhi DNA amplification kit (GE Healthcare, Piscataway, NJ) to yield high-quality DNA sufficient for single nucleotide polymorphism (SNP) genotyping.

### Single nucleotide polymorphism genotyping

DNA was genotyped using Taqman SNP allelic discrimination on the ABI 7900 HT (Applied Biosystems, 850 Lincoln Centre Drive, Foster City, CA 94404 USA) using published primers [8,9,18,46]. We studied only the *CTLA-4 *CT60 (rs3087243) allele. We chose the PADI4_94 allele (rs2240340) of the haplotype first described by Suzuki and coworkers [8], because it had the strongest association in a Japanese population and was replicated in a large meta-analysis [25]. Using the same methods, we also genotyped the lactase gene (rs4988235), which is known to exhibit substantial variation in allele frequency from Northern to Southern Europe, in order to test for population stratification in this nested case-control study [47,48].

### *HLA-DRB1 *shared epitope determination

Low-resolution HLA-DRB1 genotyping was performed by polymerase chain reaction with sequence specific primers using OLERUP SSP kits (QIAGEN, West Chester, PA, USA). We used primers to amplify DNA samples that contained sequences for HLA-DRB1*04, *01,*10 and *14, along with consensus primers and appropriate positive and negative control samples. For samples with positive two-digit HLA signals, sequence-specific primers were used for high resolution four-digit shared epitope allele detection of DRB1*0401, *0404, *0405, *0101, *0102, *1402, and *1001. OLERUP SSP computer software (QIAGEN) was used to determine four-digit HLA types. Quality control split samples were included, randomly interspersed with study samples.

### Covariate information

Information was collected from the women in both cohorts via biennial questionnaires regarding diseases, lifestyle, and health practices. Age was updated in each cycle. Reproductive covariates were chosen based on our past findings of associations between reproductive factors and risk for developing RA in this cohort [41]. Data on parity, total duration of breast-feeding, menopausal status, and postmenopausal hormone use were selected from the questionnaire cycle before the date of RA diagnosis (or index date in controls). Self-reported menopausal status and age at menopause are highly reproducible in our cohorts; in a validation study of a subsample of NHS participants, 82% of naturally postmenopausal women reported the same age at menopause (within 1 year) on two questionnaires mailed 2 years apart [49].

Participants in both cohorts were asked at baseline whether they were a current smoker or had ever smoked in the past and the age at which they began to smoke. Current smokers were asked for the number of cigarettes typically smoked per day and former smokers reported the age at which they stopped smoking and the number of cigarettes smoked per day before quitting. On each subsequent questionnaire, participants reported whether they currently smoked and the number of cigarettes smoked per day. From these reports, we calculated pack years of smoking (product of years of smoking and packs of cigarettes per day).

Other potential confounders examined included, body mass index, which was computed for each 2-year time interval using the most recent weight (in kilograms) divided by height (in meters squared), as reported at baseline. Alcohol intake was reported at least every 4 years and categorized in grams per day. Husband's educational level was assessed in 1992 in NHS and 1999 in NHSII, and was included as a proxy for socioeconomic level.

### Statistical analyses

We verified Hardy-Weinberg equilibrium for each of the genotypes among controls in each of the datasets (NHS blood, NHS cheek cells, and NHSII blood). We employed conditional logistic regression analyses, conditioned on matching factors, and adjusted for potential confounders, including cigarette smoking and reproductive factors assessed before diagnosis of RA. All analyses were first conducted separately in each cohort and then on data pooled from the two cohorts. Because the *P *value for heterogeneity was significant for the *CTLA-4 *genotype, we also meta-analytically pooled results from the two cohorts using a DerSimonian and Laird random effects model [50]. In analyses stratified by the presence of RF among the RA cases, we employed unconditional logistic regression analyses, adjusting for each of the matching factors, in addition to the covariates above. For analyses of *PTPN22*, we employed a dominant model because the minor allele frequencies were low (9% in controls and 14% in cases). In analyses involving *CTLA-4 *and *PADI-4*, we assessed the risk for RA in dominant, additive, and recessive models.

### Gene-environment and gene-gene interactions

We conducted assessments for gene-environment interactions by testing for both additive and multiplicative interactions. For additive interactions, we calculated the attributable proportion due to interaction using a 2 × 2 factorial design to analyze the data [51-53]. (There is evidence of interaction when the attributable proportion is not equal to 0.) Ninety-five per cent confidence intervals (CIs) were calculated using the delta method as described by Hosmer and Lemeshow [54]. We tested for multiplicative interaction using an interaction variable (for example, gene × smoking) in the conditional logistic regression models. The significance of the interaction was determined using the Wald χ^2 ^test of the interaction variable. In the combined NHS-NHSII nested case-control study dataset, we assessed for interactions between the presence of each polymorphism and cigarette smoking categorized both as ever/never, and then dichotomized as ≤10 or ≥10 pack-years of smoking, because this is the threshold we previously identified to be associated with increased risk for RA [35]. Using similar methods, we tested for gene-gene interaction between *PTPN22 *and *HLA-SE *in influencing RA susceptibility in analyses limited to NHS and NHSII blood samples. SAS version 9.1 (SAS Institute, Cary, NC, USA) was used for all analyses.

## Results

A total of 437 pairs of Caucasian women, each containing one woman with incident RA and her matched control, were included in these analyses, after removing 18 women because of missing data for all genotypes examined. The characteristics of the RA cases at diagnosis in each of the two cohorts are shown in Table 1. The cases in the NHS had a mean (± standard deviation) age of 57 years (± 9), as compared with 43 (± 5) in the younger NHSII cohort, because of the different ages targeted for enrollment in the two cohorts. Otherwise, the cases were similar in terms of the prevalence of RF, erosions, nodules, and proportion diagnosed by a member of the ACR. All cases and controls in these analyses were Caucasian, and the mean (± standard deviation) number of ACR criteria for the classification of RA was 5 (± 1) [43].

Table 2 shows the characteristics of the RA cases and matched controls at the time of RA diagnosis (or index date for the controls). A higher proportion of RA cases and controls were postmenopausal at RA diagnosis in the NHS than in the NHSII cohort, but the proportions of premenopausal and postmenopausal women among cases and controls were similar in each of the cohorts, as were the proportions currently receiving postmenopausal hormones. In NHSII a slightly higher percentage of women with RA were parous as compared with their matched controls (94% and 86%), but this was not true in the NHS cohort (91% of RA cases and 95% of controls). Among women in the NHSII with RA, a higher proportion had husbands who were college educated as compared with their matched controls (39% compared to 18%), but this was not true in the NHS cohort (20% in each group). No significant differences in allele frequencies of the lactase gene (rs4988235) in cases compared with controls were found. This argues strongly against any significant population stratification in our samples.

**Table 1:** Characteristics of RA cases at diagnosis of RA

Characteristic	NHS (*n* = 388)	NHSII (*n* = 49)
Age at diagnosis (mean ± SD)	57 ± 9	43 ± 5
Rheumatoid factor positive (*n *[%])	223 (57%)	27 (55%)
Rheumatoid nodules (*n *[%])	51 (13%)	6 (12%)
Radiographic changes (*n *[%])	117 (30%)	17 (35%)
Diagnosed by a member of ACR (*n *[%])	320(84%)	47 (96%)
Mean number of ACR criteria for RA [44], (SD)	5 ± 1	5 ± 1

ACR, American College of Rheumatology; NHS, Nurses' Health Study; RA, rheumatoid arthritis; SD, standard deviation.

**Table 2:** Characteristics of RA cases and matched controls: Caucasian matched pairs

Characteristics	NHS (388 matched pairs)		NHSII (49 matched pairs)	
	
	RA cases	Controls	RA cases	Controls
Matching factors				
Age (years; mean ± SD)	57 ± 9	57 ± 9	43 ± 5	43 ± 5
Postmenopausal (*n *[%])	263 (68%)	255 (66%)	14 (29%)	14 (29%)
Current PMH use (*n *[%])^a^	111 (36%)	101 (34%)	11 (69%)	12 (75%)
Other characteristics				
Ever cigarette smokers (*n *[%])	246 (63%)	220 (57%)	19 (39%)	22 (45%)
Pack-years among smokers, mean (SD)	24 ± 17	24 ± 20	16 ± 9	11 ± 6
Parous (*n *[%])	352 (91%)	367 (95%)	46 (94%)	42 (86%)
Breastfed babies ≥ 12 months total, among parous (*n *[%])^a^	44 (13%)	65 (18%)	18 (43%)	18 (45%)
Age at menarche <12 years (*n *[%])	87 (22%)	77 (20%)	16 (33%)	15 (31%)
Irregular menstrual cycle (*n *[%])	55 (14%)	49 (13%)	9 (18%)	11 (22%)
Husband education > college graduate (*n *[%])	77 (20%)	77 (20%)	19 (39%)	9 (18%)
Physical activity (hours/day mean ± SD)	2 ± 3	2 ± 5	2 ± 3	3 ± 3
Alcohol intake (g/day; mean ± SD)	6 ± 10	6 ± 9	2 ± 1	2 ± 2
Body mass index (kg/m^2^; mean ± SD)	25 ± 4	26 ± 5	27 ± 7	26 ± 5

^a^Percentage is calculated among postmenopausal women or parous women, with unknown/missing group excluded. For the rest of variables, percentage was calculated with missing category included as a separate category. NHS, Nurses' Health Study; PMH, postmenopausal hormone; RA, rheumatoid arthritis; SD, standard deviation.

The genotype and allele frequencies of the RA cases and controls for the three candidate genotypes are shown in Table 3. None of the *PTPN22*, *CTLA-4*, or *PADI-4 *genotype distributions deviated from Hardy-Weinberg equilibrium, either in each cohort or in the combined dataset. Overall, genotyping call rates were 97.5% for *PTPN22*, 96.4% for *CTLA-4*, 97.9% for *PADI-4*, and 98.7% for *HLA-SE*. The frequency of the T allele of the *PTPN22 *polymorphism was significantly higher among RA cases than among controls (χ^2 ^with one degree of freedom, *P *= 0.001 for pooled NHS and NHSII cohorts). The mutant alleles were not statistically associated with RA case status for the other two genotypes, namely *PADI-4 *and *CTLA-4*. As expected, *HLA-SE *alleles were highly significantly associated with risk for RA. (A slightly higher frequency of NHS cheek cell DNA samples could not be HLA genotyped: 3% of cases and controls, as compared with 0% to 2% of NHS and NHSII case and control DNA samples from blood.)

**Table 3:** Genotype and allele frequencies of the RA cases and controls for the three candidate genotypes (*PTPN22*, *CTLA-4*, and *PADI-4*) and genotype frequencies of *HLA-SE*

Gene	Genotype/allele	NHS blood (219 matched pairs)		NHS cheek cell (169 matched pairs)		NHSII blood (49 matched pairs)	
		
		Cases	Controls	Cases	Controls	Cases	Controls
*PTPN22*^a^	Genotype						
	CC	160 (74%)	178 (82%)	125 (77%)	131 (80%)	37 (79%)	44 (94%)
	CT	50 (23%)	38 (18%)	30 (18%)	31 (19%)	7 (15%)	3 (6%)
	TT	5 (2%)	1 (0.5%)	8 (5%)	1 (1%)	3 (6%)	0 (0%)
	Missing	4	2	6	6	2	2
	*P*^e^	0.08		0.07		0.06	
	Allele						
	C	370 (86%)	394 (91%)	280 (86%)	293 (90%)	81 (86%)	91 (97%)
	T	60 (14%)	40 (9%)	46 (14%)	33 (10%)	13 (14%)	3 (3%)
	*P*^f^	0.03		0.12		0.009	
*CTLA-4*^b^	Genotype						
	AA	35 (17%)	46 (22%)	40 (25%)	29 (18%)	7 (15%)	12 (25%)
	AG	106 (50%)	96 (45%)	69 (42%)	80 (50%)	26 (54%)	19 (40%)
	GG	71 (33%)	70 (33%)	54 (33%)	51 (32%)	15 (31%)	17 (35%)
	Missing	7	7	6	9	1	1
	*P*^f^	0.37		0.27		0.28	
	Allele						
	A	176 (42%)	188 (44%)	149 (46%)	138 (43%)	40 (42%)	43 (45%)
	G	248 (58%)	236 (56%)	177 (54%)	182 (57%)	56 (58%)	53 (55%)
	*P*^f^	0.41		0.51		0.66	
*PADI-4*^c^	Genotype						
	GG	76 (35%)	76 (36%)	57 (35%)	64 (39%)	22 (45%)	18 (38%)
	GA	107 (49%)	101 (47%)	68 (41%)	73 (44%)	16 (33%)	21 (45%)
	AA	34 (16%)	37 (17%)	39 (24%)	28 (17%)	11 (22%)	8 (17%)
	Missing	2	5	5	4	0	2
	*P*^f^	0.87		0.30		0.47	
	Allele						
	G	259 (60%)	253 (59%)	182 (55%)	201 (61%)	60 (61%)	57 (61%)
	A	175 (40%)	175 (41%)	146 (45%)	129 (39%)	38 (39%)	37 (39%)
	*P*^f^	0.87		0.16		0.93	
*HLA-SE*^d^	0 copy	84 (38%)	122 (56%)	99 (60%)	114 (70%)	23 (47%)	30 (63%)
	1 copies	99 (45%)	83 (38%)	52 (32%)	40 (24%)	17 (35%)	17 (35%)
	2 copies	36 (16%)	14 (6%)	13 (8%)	10 (6%)	9 (18%)	1 (2%)
	Missing	0	0	5	5	0	1
	*P*^f^	0.0001		0.22		0.03	

^a^Unable to genotype 12 cases and 10 controls. ^b^Unable to genotype 14 cases and 17 controls. ^c^Unable to genotype 7 cases and 11 controls. ^d^Unable to genotype 5 cases and 5 controls. ^e^*P *values estimated from the Fisher's exact test. ^f^*P *values estimated from the χ^2 ^test. NHS, Nurses' Health Study; RA, rheumatoid arthritis. OK

Table 4 includes the results of conditional logistic regression analyses of risk for RA associated with each of the genotypes, performed separately in each cohort, and then on pooled data. The final multivariable model includes pack-years of cigarette smoking, age at menarche, regularity of menses, parity, and total duration of breast-feeding. Further adjustment for body mass index, alcohol intake, husband's educational level, and oral contraceptive use did not affect risk estimates either and these were not included in the final models. As is evident comparing the results of a model taking only the matching factors into account, adjustment for potential confounders did not significantly influence results. The risk for RA associated with the *PTPN22 *variant T allele was elevated (OR = 1.46 [95% CI = 1.02 to 2.08] in a NHS and NHSII pooled multivariable dominant model). These results may have been influenced by the high OR observed in the smaller NHSII cohort (OR = 8.77). The *CTLA-4 *variant G allele was associated with an increased RA risk among women in the NHS cohort (multivariable dominant model OR = 1.92 [95% CI = 1.10 to 3.35]), but not in the NHSII cohort or pooled results (multivariable dominant model OR = 1.27 [95% CI = 0.88 to 1.84]; the OR was similar [1.29, 95% CI = 0.54 to 3.08] in a random effects meta-analytically pooled dominant model). The *PADI-4 *genotype was not associated with risk for RA in the NHS and NHSII cohorts in any of the models.

**Table 4:** Effect of *PTPN22*, *CTLA-4*, and *PADI-4 *genotypes and the risk of RA in NHS, NHSII and pooled Caucasian matched pairs

Gene	Model	Genotype	NHS blood	NHS cheek	NHSII blood	NHS and NHSII pooled		
			
			OR^a ^(95% CI)	OR^a ^(95% CI)	OR^a ^(95% CI)	OR^b ^(95% CI)	OR^a ^(95% CI)	*P *for heterogeneity^c^
*PTPN22*^d^	Dominant	CC	1.00 (ref.)	1.00 (ref.)	1.00 (ref.)	1.00 (ref.)	1.00 (ref.)	
		CT/TT	1.59 (0.96–2.64)	1.10 (0.62–1.96)	8.77 (1.11–69.51)	1.47 (1.05–2.05)	1.46 (1.02–2.08)	0.14
*CTLA-4*	Dominant	AA	1.00 (ref.)	1.00 (ref.)	1.00 (ref.)	1.00 (ref.)	1.00 (ref.)	
		AG/GG	1.92 (1.10–3.35)	0.61 (0.32–1.15)	2.18 (0.65–7.38)	1.12 (0.79–1.58)	1.27 (0.88–1.84)	0.02
	Additive	AA	1.00 (ref.)	1.00 (ref.)	1.00 (ref.)	1.00 (ref.)	1.00 (ref.)	
		AG	1.98 (1.09–3.59)	0.52 (0.26–1.04)	2.43 (0.68–8.67)	1.09 (0.76–1.58)	1.24 (0.84–1.84)	0.01
		GG	1.85 (1.00–3.41)	0.82 (0.38–1.77)	1.71 (0.40–7.31)	1.15 (0.78–1.70)	1.31 (0.86–1.99)	0.26
	Recessive	AA/AG	1.00 (ref.)	1.00 (ref.)	1.00 (ref.)	1.00 (ref.)	1.00 (ref.)	
		GG	1.13 (0.74–1.74)	1.31 (0.73–2.37)	0.89 (0.30–2.63)	1.08 (0.81–1.44)	1.12 (0.83–1.52)	0.82
*PADI-4*	Dominant	GG	1.00 (ref.)	1.00 (ref.)	1.00 (ref.)	1.00 (ref.)	1.00 (ref.)	
		GA/AA	1.10 (0.71–1.71)	1.13 (0.69–1.86)	0.48 (0.15–1.58)	1.02 (0.77–1.35)	1.04 (0.77–1.40)	0.41
	Additive	GG	1.00 (ref.)	1.00 (ref.)	1.00 (ref.)	1.00 (ref.)	1.00 (ref.)	
		GA	1.11 (0.70–1.75)	0.89 (0.51–1.55)	0.19 (0.04–1.03)	0.97 (0.72–1.32)	0.96 (0.70–1.33)	0.14
		AA	1.07 (0.56–2.05)	1.72 (0.88–3.36)	1.07 (0.23–4.89)	1.14 (0.77–1.68)	1.24 (0.82–1.89)	0.58
	Recessive	GG/GA	1.00 (ref.)	1.00 (ref.)	1.00 (ref.)	1.00 (ref.)	1.00 (ref.)	
		AA	1.00 (0.56–1.77)	1.82 (0.98–3.38)	1.51 (0.38–5.96)	1.16 (0.81–1.65)	1.27 (0.87–1.86)	0.37

^a^Conditional logistic regression, adjusting for pack-year smoking, parity, breast-feeding, menstrual irregularity, and age at menarche. ^b^Conditional logistic regression. ^c^*P *for heterogeneity was calculated using multivariable model a for each cohort. ^d^A dominant model only was tested for the *PTPN22 *genotype given a low homozygous risk genotype of 2%. CI, confidence interval; NHS, Nurses' Health Study; OR, odds ratio; RA, rheumatoid arthritis.

To pursue potential associations of these polymorphisms with different RA phenotypes, we conducted analyses stratified by RF positivity, because many risk factors, including *HLA-SE *and cigarette smoking, have been shown to be more strongly associated with RF-seropositive RA [35,36]. Results of these analyses are shown in Table 5. The effect of the *PTPN22 *polymorphism was seen primarily for the development of RF-seropositive RA (OR = 1.75 [95% CI = 1.18 to 2.59]).

Cigarette smoking is a strong environmental risk factor for the development of RA, in particular RF-positive RA, and amount and duration are associated with increased risk [35]. We thus investigated potential interactions between the three polymorphisms of interest and the amount and duration of cigarette smoking at the time of RA diagnosis. Table 6 presents the results of analyses in which we tested for both multiplicative and additive interactions between smoking, categorized as ever/never smoking and then dichotomized as ≤10 or ≥10 pack-years of smoking, for each of the genotypes. Among those with the CC genotype of *PTPN22*, a modest effect of heavy smoking was observed (OR = 1.22 [95% CI = 0.81 to 1.83). However, among those with the *PTPN22 *T risk allele, the effect of heavy smoking was much more pronounced (OR = 2.50 [95% CI = 1.25 to 5.00]). We observed significant additive and multiplicative gene-environment interactions between heavy cigarette smoking and the presence of the *PTPN22 *T allele (additive interaction: *P *= 0.0006; multiplicative interaction: *P *= 0.04). When smoking was dichotomized as never/ever, there was marginal evidence for additive but not multiplicative interaction. We also tested for genotype-smoking interactions in RF-positive and RF-negative RA cases separately. In stratified analyses, we found significant additive but not multiplicative interactions between the *PTPN22 *risk allele and heavy smoking for both seropositive and seronegative RA. We did not observe similar gene-smoking interactions for *CTLA-4 *or *PADI-4*, for the overall risk for RA, or for RF-positive or RF-negative RA separately. No additive or multiplicative interactions were observed between *PTPN22 *and *HLA-SE *(Table 7). (Given potential difficulties with *HLA-SE *genotyping NHS cheek cell DNA samples, we performed sensitivity analyses with these samples excluded, and the interaction analyses yielded similar and nonsignificant findings.)

**Table 5:** Stratified analyses of genotype and RA risk in the pooled NHS/NHSII samples

Gene/genotype	RF status of RA cases (versus controls)	
	
	RF-positive cases	RF-negative cases
*PTPN22*		
CC	1.00 (ref.)	1.00 (ref.)
CT/TT	1.75 (1.18–2.59)	1.32 (0.85–2.06)
*CTLA-4*		
AA	1.00 (ref.)	1.00 (ref.)
AG/GG	1.18 (0.78–1.80)	1.11 (0.71–1.72)
*PADI-4*		
GG	1.00 (ref.)	1.00 (ref.)
GA/AA	1.10 (0.79–1.54)	0.96 (0.66–1.38)

The analysis was unconditional logistic regression adjusting for year of birth, pack-year smoking, parity, breast-feeding, menstrual irregularity, age at menarche, menopausal status and postmenopausal hormone use. Values are expressed as odds ratio (95% confidence interval). CI, confidence interval; NHS, Nurses' Health Study; OR, odds ratio; RA, rheumatoid arthritis; RF, rheumatoid factor.

**Table 6:** *PTPN22 *genotype and smoking interactions according to RF status in NHS/NHSII pooled samples

Smoking status/pack-years	*PTPN22 *Genotype	All cases^a^	RF-positive cases^b,c^	RF-negative cases^b,d^
Smoking status				
Never	CC	1.00 (ref.)	1.00 (ref.)	1.00 (ref.)
Ever	CC	1.10 (0.79–1.53)	1.37 (0.93–2.01)	1.02 (0.68–1.53)
Never	CT/TT	1.07 (0.61–1.86)	1.57 (0.86–2.87)	0.87 (0.43–1.77)
Ever	CT/TT	1.90 (1.14–3.16)	2.56 (1.47–4.46)	1.67 (0.91–3.06)
Additive interaction		AP = 0.39 (95% CI -0.04 to +0.82); *P*_add_^e ^= 0.08	AP = 0.24 (95% CI -0.26 to +0.75); *P*_add_^e ^= 0.35	AP = 0.47 (95% CI -0.03 to +0.96); *P*_add_^e ^= 0.07
Multiplicative interaction		*P*_multi_^f ^= 0.20	*P*_multi_^f ^= 0.67	*P*_multi_^f ^= 0.18
Pack-years smoking				
<10	CC	1.00 (ref.)	1.00 (ref.)	1.00 (ref.)
>10	CC	1.25 (0.89–1.75)	1.40 (0.96–2.05)	1.22 (0.81–1.83)
<10	CT/TT	1.09 (0.69–1.73)	1.48 (0.89–2.47)	1.00 (0.55–1.82)
>10	CT/TT	3.05 (1.64–5.66)	3.27 (1.74–6.15)	2.50 (1.25–5.00)
Additive interaction		AP = 0.56 (95% CI 0.24 to 0.88); *P*_add_^e ^= 0.0006	AP = 0.42 (95% CI 0.01 to 0.84); *P*_add_^e ^= 0.04	AP = 0.51 (95% CI 0.09 to 0.93); *P*_add_^e ^= 0.02
Multiplicative interaction		*P*_multi_^f ^= 0.04	*P*_multi_^f ^= 0.28	*P*_multi_^f ^= 0.13

Values are expressed as odds ratio (95% confidence interval). ^a^Conditional logistic regression, adjusting for parity, breast-feeding, menstrual irregularity, and age at menarche, menopausal status and postmenopausal hormone use. ^b^Unconditional logistic regression adjusting for year of birth, parity, breast-feeding, menstrual irregularity, and age at menarche, menopausal status and postmenopausal hormone use. ^c^RF-positive RA cases and all controls. ^d^RF-negative RA cases and all controls. ^e^*P*_add _is the *P *value for attributable proportion (AP), one of the indices of additive interactions between binary smoking variable and binary *PTPN22 *genotype. ^f^*P*_multi _is the *P *value for multiplicative interaction term between binary smoking variable and binary *PTPN22 *genotype with one degree of freedom. NHS, Nurses' Health Study; RA, rheumatoid arthritis; RF, rheumatoid factor.

**Table 7:** *PTPN22 *genotype and *HLA-SE *interactions according to RF status in NHS/NHSII pooled samples

*PTPN22*	*HLA-SE*	All cases^a^	RF-positive cases^b,c^	RF-negative cases^b,d^
CC	None	1.00 (ref.)	1.00 (ref.)	1.00 (ref.)
CC	Any	1.97 (1.39–2.78)	2.37 (1.62–3.46)	1.33 (0.89–1.99)
T carrier	None	1.41 (0.87–2.27)	1.76 (0.99–3.13)	1.40 (0.78–2.51)
T carrier	Any	2.73 (1.55–4.81)	3.58 (2.02–6.35)	1.47 (0.74–2.92)
Additive interaction		AP = 0.13 (95% CI -0.40 to +0.66); *P*_add_^e ^= 0.62	AP = 0.13 (95% CI -0.41 to +0.67); *P*_add_^e ^= 0.64^e^	AP = -0.18^f^
Multiplicative interaction		*P*_multi_^g ^= 0.97^g^	*P*_multi_^g ^= 0.72	*P*_multi_^g ^= 0.60

Values are expressed as odds ratio (95% confidence interval). ^a^Conditional logistic regression, adjusting for parity, pack-year smoking, breast-feeding, menstrual irregularity, and age at menarche, menopausal status and postmenopausal hormone use. ^b^Unconditional logistic regression adjusting for year of birth, parity, pack-year smoking, breast-feeding, menstrual irregularity, and age at menarche, menopausal status and postmenopausal hormone use. ^c^RF-positive RA cases and all controls. ^d^RF-negative RA cases and all controls. ^e^*P *for AP, one of the indices of additive interactions between binary *PTPN22 *genotype and binary *HLA-SE *genotype. ^f^95% confidence interval and *P *value for AP not applicable because of the negative estimate (antagonistic rather than synergistic). ^g^*P *for multiplicative interaction term between binary *PTPN22 *genotype and binary *HLA-SE *genotype with one degree of freedom. AP, attributable proportion; NHS, Nurses' Health Study; RA, rheumatoid arthritis; RF, rheumatoid factor.

## Discussion

In these two cohorts of women followed prospectively for the development of RA and for multiple potential environmental exposures, we have confirmed that the R620W polymorphism in the *PTPN22 *gene is associated with increased risk for RA. We did not confirm that the *PADI-4 *(rs2240340) or the *CTLA-4 *(rs3087243) polymorphism were associated with increased risk for RA or for RF-positive RA in this population. We did not find that cigarette smoking, parity, total duration of breastfeeding, age at menarche, regularity of menses, menopausal status, or postmenopausal hormone use – all associated with risk for RA in past studies – were important confounders of the relationships between these genotypes and RA. However, we did uncover a significant multiplicative gene-environment interaction between heavy smoking and *PTPN22 *in determining RA risk.

The C→T polymorphism at position 1858 of the *PTPN22 *gene interferes with the function of the *PTPN22*/Csk complex, which is an important inhibitor of T-cell signaling, hindering its ability to suppress T-cell activation [9,26,55]. Similar to past reports, we have found the elevated risk to be primarily for RF-positive disease [9,25,56-58]. Several reports and a meta-analysis have suggested that those with the *PTPN22 *risk allele have more severe disease [25,57]. We have confirmed that a significant association exists after adjustment for potential confounders, including smoking and reproductive factors. We also found a significant multiplicative interaction between heavy cigarette smoking (≥10 pack-years) and the presence of the *PTPN22 *risk allele, with a threefold elevated odds of developing RA in the presence of both factors.

Kallberg and colleagues [38] recently explored potential gene-environment and gene-gene interactions in RA susceptibility, combining data from three large RA cohort studies. The results of their study are slightly different from ours, in that they did not find a significant interaction between the presence of the *PTPN22 *polymorphism and smoking in determining RA risk. Their gene-smoking interaction analyses used data from the Swedish Environmental Investigations in RA incident RA cohort, in which participants were asked to recall past smoking and were classified as ever or never smokers. Using the detailed prospective data regarding smoking amount and duration available for NHS and NHSII participants, we demonstrated a multiplicative interaction between the presence of the *PTPN22 *risk allele and heavy cigarette smoking of ≥10 pack-years in this female cohort. In past studies, we have found that the risk for RA was significantly elevated with ≥10 pack-years [35]. Our results now suggest that it may be necessary to exceed a threshold of heavy smoking to trigger a biologic pathway in RA pathogenesis involving the *PTPN22 *gene. Both *HLA-SE *and *PTPN22 *primarily affect the risk for RF-positive and anti-CCP-positive RA [25,36,59-61].

In the case of *HLA-SE*, it is hypothesized that cigarette smoking leads to inflammation and citrullination of certain peptides, which – when presented within the context of HLA-DR4 molecules – are specifically recognized, contributing to anti-citrulline autoimmunity [37]. The newly described interaction between *PTPN22 *and heavy cigarette smoking suggests that the smoking/citrullination/T-cell recognition and activation pathway in RA pathogenesis may be influenced by both *PTPN22 and HLA-SE*.

The *CTLA-4 *gene is an attractive candidate gene for RA susceptibility, given the role played by CTLA-4 (cytotoxic T-lymphocyte associated 4) in T-cell activation and that a CTLA-4-IgG1 fusion protein is very effective in treating RA [62]. The CT60 polymorphism was associated with a modest increase in RA risk in the NHS cohort alone, and not in the NHSII cohort or pooled results, possibly because of a lack of sufficient power to detect a small elevation in risk (with OR in the order of 1.2) reported in other studies [25]. In a *post hoc *power calculation, for this *CTLA-4 *genotype with a risk allele frequency of 0.56 among controls and a two-sided type I error rate of 0.05, we had 71% power to detect an effect of 40% or greater (OR = 1.4).

The enzyme peptidylarginine deiminase-4, responsible for the citrullination of peptides to which anti-CCP antibodies are formed, is encoded by the *PADI-4 *gene. The *PADI4_94 *SNP we have investigated was associated with RA in Japanese subjects (OR = 1.97 [95% CI = 1.44 to 2.69]) [8]. The effect sizes observed in previous replication studies in Caucasians have been small (pooled OR = 1.1 [95% CI = 1.0 to 1.2]) [25,.64]. We were unable to detect an effect of this polymorphism on the risk for RA in these cohorts of women, and this could reflect inadequate power to detect a risk estimate of that magnitude. Given that the allele frequencies in the controls were similar in each of the cohorts to that reported in the literature, the significant *P *value for heterogeneity across the cohorts we observed was probably due to small sample size.

Limitations of this study that should be noted include the fact that, in the NHS and NHSII cohorts, the presence or absence of RF in the blood among RA cases was confirmed by medical record review at diagnosis, and thus not assayed at the same laboratory, and was not assayed in controls. Rheumatoid nodules and radiographic erosions are likewise documented at the time of diagnosis from thorough medical record review, but cohort participants have not been followed longitudinally for RA disease activity or complications. Similarly, we have limited data in the medical record on antibodies to CCP among the cases, which is important in the subphenotyping of RA [64], because the dates of diagnosis for most of the RA cases in this cohort preceded the clinical use of anti-CCP. Further analysis by anti-CCP status could be potentially informative.

Although all participants included in this analysis were of self-reported Caucasian ancestry, potential population stratification, or confounding by ethnicity, still exists, in particular if the inclusion of individuals of Northern compared with Southern European origin varied between cases and controls [48,65,66]. We assessed the potential for this bias in two ways. First, we examined and did not find significant differences in the more precise racial backgrounds reported by the Caucasian women included as cases or controls in these analyses. Second, we genotyped the lactase gene, which is known to exhibit substantial variation in allele frequency from Northern to Southern Europe [47,48], and found no significant differences in allele frequencies between cases and controls. A recent whole-genome association study investigating breast cancer risk alleles [67] found no evidence of population stratification among self-reported Caucasian women in the NHS cohort.

This study is unique in that the participants were followed for many years, in great detail, before the onset on their RA, and environmental and reproductive risk factors for RA have been well studied in this cohort [35,41]. This has allowed the investigation of possible gene-environment interactions with each of these recently described polymorphisms, and known and suspected RA risk factors assessed prospectively, such as cigarette smoking and menopausal status.

## Conclusion

Our data confirm that the *PTPN2*2 R620W polymorphism is a strong risk factor for RF-positive RA, and that presence of this polymorphism interacts with heavy cigarette smoking in a multiplicative manner. These findings contribute to the growing understanding of how genetic and environmental factors interact in RA pathogenesis, and suggest that heavy cigarette smoking and *PTPN22 *may be acting in a similar mechanistic pathway.

## Abbreviations

ACR = American College of Rheumatology; CCP = cyclic citrullinated peptide; CI = confidence interval; HLA = human leukocyte antigen; NHS = Nurses' Health Study; OR = odds ratio; RA = rheumatoid arthritis; RF = rheumatoid factor; SE = shared epitope; SNP = single nucleotide polymorphism.

## Competing interests

The authors declare that they have no competing interests.

## Authors' contributions

KHC was responsible for study design, data acquisition, analysis and interpretation of data, manuscript preparation, and statistical analysis. S-CC was responsible for analysis and interpretation of data, manuscript preparation, and statistical analysis. IDV was responsible for analysis and interpretation of data, manuscript preparation, and statistical analysis. RP was responsible for study design, analysis and interpretation of data, and manuscript preparation. EWK was responsible for study design, data acquisition, analysis and interpretation of data, manuscript preparation, and statistical analysis.

## References

[B1] Gabriel SE, Crowson CS, O'Fallon WM (1999). The epidemiology of rheumatoid arthritis in Rochester, Minnesota, 1955–1985. Arthritis Rheum.

[B2] Cornélis F, Fauré S, Martinez M, Prud'homme JF, Fritz P, Dib C, Alves H, Barrera P, de Vries N, Balsa A, Pascual-Salcedo D, Maenaut K, Westhovens R, Migliorini P, Tran TH, Delaye A, Prince N, Lefevre C, Thomas G, Poirier M, Soubigou S, Alibert O, Lasbleiz S, Fouix S, Bouchier C, Lioté F, Loste MN, Lepage V, Charron D, Gyapay G (1998). New susceptibility locus for rheumatoid arthritis suggested by a genome-wide linkage study. Proc Natl Acad Sci USA.

[B3] Jawaheer D, Gregersen PK (2002). Rheumatoid arthritis. The genetic components. Rheum Dis Clin North Am.

[B4] Gregersen PK, Silver J, Winchester RJ (1987). The shared epitope hypothesis. An approach to understanding the molecular genetics of susceptibility to rheumatoid arthritis. Arthritis Rheum.

[B5] Thomson W, Harrison B, Ollier B, Wiles N, Payton T, Barrett J, Symmons D, Silman A (1999). Quantifying the exact role of HLA-DRB1 alleles in susceptibility to inflammatory polyarthritis: results from a large, population-based study. Arthritis Rheum.

[B6] Jawaheer D, Seldin MF, Amos CI, Chen WV, Shigeta R, Etzel C, Damle A, Xiao X, Chen D, Lum RF, Monteiro J, Kern M, Criswell LA, Albani S, Nelson JL, Clegg DO, Pope R, Schroeder HW, Bridges SL, Pisetsky DS, Ward R, Kastner DL, Wilder RL, Pincus T, Callahan LF, Flemming D, Wener MH, Gregersen PK (2003). Screening the genome for rheumatoid arthritis susceptibility genes: a replication study and combined analysis of 512 multicase families. Arthritis Rheum.

[B7] Gonzalez-Escribano MF, Rodriguez R, Valenzuela A, Garcia A, Garcia-Lozano JR, Nunez-Roldan A (1999). CTLA4 polymorphisms in Spanish patients with rheumatoid arthritis. Tissue Antigens.

[B8] Suzuki A, Yamada R, Chang X, Tokuhiro S, Sawada T, Suzuki M, Nagasaki M, Nakayama-Hamada M, Kawaida R, Ono M, Ohtsuki M, Furukawa H, Yoshino S, Yukioka M, Tohma S, Matsubara T, Wakitani S, Teshima R, Nishioka Y, Sekine A, Iida A, Takahashi A, Tsunoda T, Nakamura Y, Yamamoto K (2003). Functional haplotypes of PADI4, encoding citrullinating enzyme peptidylarginine deiminase 4, are associated with rheumatoid arthritis. Nat Genet.

[B9] Begovich AB, Carlton VE, Honigberg LA, Schrodi SJ, Chokkalingam AP, Alexander HC, Ardlie KG, Huang Q, Smith AM, Spoerke JM, Conn MT, Chang M, Chang SY, Saiki RK, Catanese JJ, Leong DU, Garcia VE, McAllister LB, Jeffery DA, Lee AT, Batliwalla F, Remmers E, Criswell LA, Seldin MF, Kastner DL, Amos CI, Sninsky JJ, Gregersen PK (2004). A missense single-nucleotide polymorphism in a gene encoding a protein tyrosine phosphatase (PTPN22) is associated with rheumatoid arthritis. Am J Hum Genet.

[B10] Radstake TR, Franke B, Hanssen S, Netea MG, Welsing P, Barrera P, Joosten LA, van Riel PL, Berg WB van den (2004). The Toll-like receptor 4 Asp299Gly functional variant is associated with decreased rheumatoid arthritis disease susceptibility but does not influence disease severity and/or outcome. Arthritis Rheum.

[B11] Criswell LA, Pfeiffer KA, Lum RF, Gonzales B, Novitzke J, Kern M, Moser KL, Begovich AB, Carlton VE, Li W, Lee AT, Ortmann W, Behrens TW, Gregersen PK (2005). Analysis of families in the Multiple Autoimmune Disease Genetics Consortium (MADGC) collection: the PTPN22 620W allele associates with multiple autoimmune phenotypes. Am J Hum Genet.

[B12] Lee AT, Li W, Liew A, Bombardier C, Weisman M, Massarotti EM, Kent J, Wolfe F, Begovich AB, Gregersen PK (2005). The PTPN22 R620W polymorphism associates with RF positive rheumatoid arthritis in a dose-dependent manner but not with HLA-SE status. Genes Immun.

[B13] Orozco G, Sanchez E, Gonzalez-Gay MA, Lopez-Nevot MA, Torres B, Caliz R, Ortego-Centeno N, Jimenez-Alonso J, Pascual-Salcedo D, Balsa A, de Pablo R, Nunez-Roldan A, Gonzalez-Escribano MF, Martin J (2005). Association of a functional single-nucleotide polymorphism of PTPN22, encoding lymphoid protein phosphatase, with rheumatoid arthritis and systemic lupus erythematosus. Arthritis Rheum.

[B14] Viken MK, Amundsen SS, Kvien TK, Boberg KM, Gilboe IM, Lilleby V, Sollid LM, Forre OT, Thorsby E, Smerdel A, Lie BA (2005). Association analysis of the 1858C>T polymorphism in the PTPN22 gene in juvenile idiopathic arthritis and other autoimmune diseases. Genes Immun.

[B15] Pierer M, Kaltenhauser S, Arnold S, Wahle M, Baerwald C, Hantzschel H, Wagner U (2006). Association of PTPN22 1858 single-nucleotide polymorphism with rheumatoid arthritis in a German cohort: higher frequency of the risk allele in male compared to female patients. Arthritis Res Ther.

[B16] Consortium WTCC (2007). Genome-wide association study of 14,000 cases of seven common diseases and 3,000 shared controls. Nature.

[B17] Vaidya B, Pearce SH, Charlton S, Marshall N, Rowan AD, Griffiths ID, Kendall-Taylor P, Cawston TE, Young-Min S (2002). An association between the CTLA4 exon 1 polymorphism and early rheumatoid arthritis with autoimmune endocrinopathies. Rheumatology (Oxford).

[B18] Rodriguez MR, Nunez-Roldan A, Aguilar F, Valenzuela A, Garcia A, Gonzalez-Escribano MF (2002). Association of the CTLA4 3' untranslated region polymorphism with the susceptibility to rheumatoid arthritis. Hum Immunol.

[B19] Remmers EF, Plenge RM, Lee AT, Graham RR, Hom G, Behrens TW, de Bakker PI, Le JM, Lee HS, Batliwalla F, Li W, Masters SL, Booty MG, Carulli JP, Padyukov L, Alfredsson L, Klareskog L, Chen WV, Amos CI, Criswell LA, Seldin MF, Kastner DL, Gregersen PK (2007). STAT4 and the risk of rheumatoid arthritis and systemic lupus erythematosus. N Engl J Med.

[B20] Plenge RM, Seielstad M, Padyukov L, Lee AT, Remmers EF, Ding B, Liew A, Khalili H, Chandrasekaran A, Davies LR, Li W, Tan AK, Bonnard C, Ong RT, Thalamuthu A, Pettersson S, Liu C, Tian C, Chen WV, Carulli JP, Beckman EM, Altshuler D, Alfredsson L, Criswell LA, Amos CI, Seldin MF, Kastner DL, Klareskog L, Gregersen PK (2007). TRAF1-C5 as a risk locus for rheumatoid arthritis: a genomewide study. N Engl J Med.

[B21] Hinks A, Barton A, John S, Bruce I, Hawkins C, Griffiths CE, Donn R, Thomson W, Silman A, Worthington J (2005). Association between the PTPN22 gene and rheumatoid arthritis and juvenile idiopathic arthritis in a UK population: further support that PTPN22 is an autoimmunity gene. Arthritis Rheum.

[B22] Prescott NJ, Fisher SA, Onnie C, Pattni R, Steer S, Sanderson J, Forbes A, Lewis CM, Mathew CG (2005). A general autoimmunity gene (PTPN22) is not associated with inflammatory bowel disease in a British population. Tissue Antigens.

[B23] Zhernakova A, Eerligh P, Wijmenga C, Barrera P, Roep BO, Koeleman BP (2005). Differential association of the PTPN22 coding variant with autoimmune diseases in a Dutch population. Genes Immun.

[B24] Kokkonen H, Johansson M, Innala L, Eriksson C, Jidell E, Rantapaa Dahlqvist S (2007). The PTPN22 1858C/T polymorphism is associated with anti-cyclic citrullinated peptide antibody positive early rheumatoid arthritis in northern Sweden. Arthritis Res Ther.

[B25] Plenge RM, Padyukov L, Remmers EF, Purcell S, Lee AT, Karlson EW, Wolfe F, Kastner DL, Alfredsson L, Altshuler D, Gregersen PK, Klareskog L, Rioux JD (2005). Replication of putative candidate-gene associations with rheumatoid arthritis in >4,000 samples from North America and Sweden: association of susceptibility with PTPN22, CTLA4, and PADI4. Am J Hum Genet.

[B26] Bottini N, Musumeci L, Alonso A, Rahmouni S, Nika K, Rostamkhani M, MacMurray J, Meloni GF, Lucarelli P, Pellecchia M, Eisenbarth GS, Comings D, Mustelin T (2004). A functional variant of lymphoid tyrosine phosphatase is associated with type I diabetes. Nat Genet.

[B27] Zheng W, She JX (2005). Genetic association between a lymphoid tyrosine phosphatase (PTPN22) and type 1 diabetes. Diabetes.

[B28] Suppiah V, O'Doherty C, Heggarty S, Patterson CC, Rooney M, Vandenbroeck K (2006). The CTLA4+49A/G and CT60 polymorphisms and chronic inflammatory arthropathies in Northern Ireland. Exp Mol Pathol.

[B29] MacGregor AJ, Snieder H, Rigby AS, Koskenvuo M, Kaprio J, Aho K, Silman AJ (2000). Characterizing the quantitative genetic contribution to rheumatoid arthritis using data from twins. Arthritis Rheum.

[B30] Karlson EW, Lee IM, Cook NR, Manson JE, Buring JE, Hennekens CH (1999). A retrospective cohort study of cigarette smoking and risk of rheumatoid arthritis in female health professionals. Arthritis Rheum.

[B31] Hutchinson D, Shepstone L, Moots R, Lear JT, Lynch MP (2001). Heavy cigarette smoking is strongly associated with rheumatoid arthritis (RA), particularly in patients without a family history of RA. Ann Rheum Dis.

[B32] Mattey DL, Dawes PT, Fisher J, Brownfield A, Thomson W, Hajeer AH, Ollier WE (2002). Nodular disease in rheumatoid arthritis: association with cigarette smoking and HLA-DRB1/TNF gene interaction. J Rheumatol.

[B33] Stolt P, Bengtsson C, Nordmark B, Lindblad S, Lundberg I, Klareskog L, Alfredsson L (2003). Quantification of the influence of cigarette smoking on rheumatoid arthritis: results from a population based case-control study, using incident cases. Ann Rheum Dis.

[B34] Karlson EW, Mandl LA, Aweh GN, Grodstein F (2003). Coffee consumption and risk of rheumatoid arthritis. Arthritis Rheum.

[B35] Costenbader KH, Feskanich D, Mandl LA, Karlson EW (2006). Smoking intensity, duration, and cessation, and the risk of rheumatoid arthritis in women. Am J Med.

[B36] Padyukov L, Silva C, Stolt P, Alfredsson L, Klareskog L (2004). A gene-environment interaction between smoking and shared epitope genes in HLA-DR provides a high risk of seropositive rheumatoid arthritis. Arthritis Rheum.

[B37] Klareskog L, Stolt P, Lundberg K, Kallberg H, Bengtsson C, Grunewald J, Ronnelid J, Erlandsson Harris H, Ulfgren AK, Rantapaa-Dahlqvist S, Eklund A, Padyukov L, Alfredsson L (2006). A new model for an etiology of rheumatoid arthritis: smoking may trigger HLA-DR (shared epitope)-restricted immune reactions to autoantigens modified by citrullination. Arthritis Rheum.

[B38] Kallberg H, Padyukov L, Plenge RM, Ronnelid J, Gregersen PK, Helm-van Mil AH van der, Toes RE, Huizinga TW, Klareskog L, Alfredsson L (2007). Gene-gene and gene-environment interactions involving HLA-DRB1, PTPN22, and smoking in two subsets of rheumatoid arthritis. Am J Hum Genet.

[B39] Hernandez-Avila M, Liang MH, Willett WC, Stampfer MJ, Colditz GA, Rosner B, Chang RW, Hennekens CH, Speizer FE (1990). Exogenous sex hormones and the risk of rheumatoid arthritis. Arthritis Rheum.

[B40] Hernandez Avila M, Liang MH, Willett WC, Stampfer MJ, Colditz GA, Rosner B, Roberts WN, Hennekens CH, Speizer FE (1990). Reproductive factors, smoking, and the risk for rheumatoid arthritis. Epidemiology.

[B41] Karlson EW, Mandl LA, Hankinson SE, Grodstein F (2004). Do breast-feeding and other reproductive factors influence future risk of rheumatoid arthritis? Results from the Nurses' Health Study. Arthritis Rheum.

[B42] Karlson EW, Daltroy LH, Lew RA, Wright EA, Partridge AJ, Roberts WN, Stern SH, Straaton KV, Wacholtz MC, Grosflam JM (1995). The independence and stability of socioeconomic predictors of morbidity in systemic lupus erythematosus. Arthritis Rheum.

[B43] Arnett FC, Edworthy SM, Bloch DA, McShane DJ, Fries JF, Cooper NS, Healey LA, Kaplan SR, Liang MH, Luthra HS (1988). The American Rheumatism Association 1987 revised criteria for the classification of rheumatoid arthritis. Arthritis Rheum.

[B44] Hankinson SE, Colditz GA, Hunter DJ, Manson JE, Willett WC, Stampfer MJ, Longcope C, Speizer FE (1995). Reproductive factors and family history of breast cancer in relation to plasma estrogen and prolactin levels in postmenopausal women in the Nurses' Health Study (United States). Cancer Causes Control.

[B45] Tworoger SS, Sluss P, Hankinson SE (2006). Association between plasma prolactin concentrations and risk of breast cancer among predominately premenopausal women. Cancer Res.

[B46] Ueda H, Howson JM, Esposito L, Heward J, Snook H, Chamberlain G, Rainbow DB, Hunter KM, Smith AN, Di Genova G, Herr MH, Dahlman I, Payne F, Smyth D, Lowe C, Twells RC, Howlett S, Healy B, Nutland S, Rance HE, Everett V, Smink LJ, Lam AC, Cordell HJ, Walker NM, Bordin C, Hulme J, Motzo C, Cucca F, Hess JF (2003). Association of the T-cell regulatory gene CTLA4 with susceptibility to autoimmune disease. Nature.

[B47] Bersaglieri T, Sabeti PC, Patterson N, Vanderploeg T, Schaffner SF, Drake JA, Rhodes M, Reich DE, Hirschhorn JN (2004). Genetic signatures of strong recent positive selection at the lactase gene. Am J Hum Genet.

[B48] Campbell CD, Ogburn EL, Lunetta KL, Lyon HN, Freedman ML, Groop LC, Altshuler D, Ardlie KG, Hirschhorn JN (2005). Demonstrating stratification in a European American population. Nat Genet.

[B49] Colditz GA, Stampfer MJ, Willett WC, Stason WB, Rosner B, Hennekens CH, Speizer FE (1987). Reproducibility and validity of self-reported menopausal status in a prospective cohort study. Am J Epidemiol.

[B50] DerSimonian R, Laird N (1986). Meta-analysis in clinical trials. Control Clin Trials.

[B51] Andersson T, Alfredsson L, Kallberg H, Zdravkovic S, Ahlbom A (2005). Calculating measures of biological interaction. Eur J Epidemiol.

[B52] Ahlbom A, Alfredsson L (2005). Interaction: a word with two meanings creates confusion. Eur J Epidemiol.

[B53] Lundberg M, Fredlund P, Hallqvist J, Diderichsen F (1996). A SAS program calculating three measures of interaction with confidence intervals. Epidemiology.

[B54] Hosmer DW, Lemeshow S (1992). Confidence interval estimation of interaction. Epidemiology.

[B55] Cloutier JF, Veillette A (1999). Cooperative inhibition of T-cell antigen receptor signaling by a complex between a kinase and a phosphatase. J Exp Med.

[B56] Dieude P, Garnier S, Michou L, Petit-Teixeira E, Glikmans E, Pierlot C, Lasbleiz S, Bardin T, Prum B, Cornelis F (2005). Rheumatoid arthritis seropositive for the rheumatoid factor is linked to the protein tyrosine phosphatase nonreceptor 22-620W allele. Arthritis Res Ther.

[B57] Steer S, Lad B, Grumley JA, Kingsley GH, Fisher SA (2005). Association of R602W in a protein tyrosine phosphatase gene with a high risk of rheumatoid arthritis in a British population: evidence for an early onset/disease severity effect. Arthritis Rheum.

[B58] Wesoly J, Helm-van Mil AH van der, Toes RE, Chokkalingam AP, Carlton VE, Begovich AB, Huizinga TW (2005). Association of the PTPN22 C1858T single-nucleotide polymorphism with rheumatoid arthritis phenotypes in an inception cohort. Arthritis Rheum.

[B59] Huizinga TW, Amos CI, Helm-van Mil AH van der, Chen W, van Gaalen FA, Jawaheer D, Schreuder GM, Wener M, Breedveld FC, Ahmad N, Lum RF, de Vries RR, Gregersen PK, Toes RE, Criswell LA (2005). Refining the complex rheumatoid arthritis phenotype based on specificity of the HLA-DRB1 shared epitope for antibodies to citrullinated proteins. Arthritis Rheum.

[B60] Irigoyen P, Lee AT, Wener MH, Li W, Kern M, Batliwalla F, Lum RF, Massarotti E, Weisman M, Bombardier C, Remmers EF, Kastner DL, Seldin MF, Criswell LA, Gregersen PK (2005). Regulation of anti-cyclic citrullinated peptide antibodies in rheumatoid arthritis: contrasting effects of HLA-DR3 and the shared epitope alleles. Arthritis Rheum.

[B61] Johansson AS, Stenberg G, Widersten M, Mannervik B (1998). Structure-activity relationships and thermal stability of human glutathione transferase P1-1 governed by the H-site residue 105. J Mol Biol.

[B62] Kremer JM, Westhovens R, Leon M, Di Giorgio E, Alten R, Steinfeld S, Russell A, Dougados M, Emery P, Nuamah IF, Williams GR, Becker JC, Hagerty DT, Moreland LW (2003). Treatment of rheumatoid arthritis by selective inhibition of T-cell activation with fusion protein CTLA4Ig. N Engl J Med.

[B63] Lee YH, Rho YH, Choi SJ, Ji JD, Song GG (2007). PADI4 polymorphisms and rheumatoid arthritis susceptibility: a meta-analysis. Rheumatol Int.

[B64] Berglin E, Padyukov L, Sundin U, Hallmans G, Stenlund H, Van Venrooij WJ, Klareskog L, Dahlqvist SR (2004). A combination of autoantibodies to cyclic citrullinated peptide (CCP) and HLA-DRB1 locus antigens is strongly associated with future onset of rheumatoid arthritis. Arthritis Res Ther.

[B65] Epstein MP, Allen AS, Satten GA (2007). A simple and improved correction for population stratification in case-control studies. Am J Hum Genet.

[B66] Wacholder S, Rothman N, Caporaso N (2000). Population stratification in epidemiologic studies of common genetic variants and cancer: quantification of bias. J Natl Cancer Inst.

[B67] Hunter DJ, Kraft P, Jacobs KB, Cox DG, Yeager M, Hankinson SE, Wacholder S, Wang Z, Welch R, Hutchinson A, Wang J, Yu K, Chatterjee N, Orr N, Willett WC, Colditz GA, Ziegler RG, Berg CD, Buys SS, McCarty CA, Feigelson HS, Calle EE, Thun MJ, Hayes RB, Tucker M, Gerhard DS, Fraumeni JF, Hoover RN, Thomas G, Chanock SJ (2007). A genome-wide association study identifies alleles in FGFR2 associated with risk of sporadic postmenopausal breast cancer. Nat Genet.

